# Purpura in the Emergency Room: Two Cases of Sepsis with Cutaneous Manifestations

**DOI:** 10.7759/cureus.4976

**Published:** 2019-06-23

**Authors:** Kenneth L Frye, Brian C McMaster, Kevin J Tomecsek

**Affiliations:** 1 Emergency Medicine, Florida Hospital, Orlando, USA; 2 Emergency Medicine, AdventHealth East Orlando, Orlando, USA

**Keywords:** purpura fulminans, mrsa, bacteremia, sepsis, meningococcemia, purpura, protein c deficiency

## Abstract

Purpura fulminans is a seldom seen manifestation of sepsis in the emergency department (ED). The morbidity and mortality of sepsis have been widely studied and reported; the hallmark of treatment is early recognition and intervention. In extreme cases, sepsis can cause widespread activation of the coagulation cascade further complicating the treatment and recovery from the causative pathogen. We report two cases and their differing outcomes after presentation to the ED with similar dermatologic findings on initial physical exam.

## Introduction

Purpura fulminans is a rare, life-threatening disease state, classically defined as a cutaneous marker of disseminated intravascular coagulation, which can be present in both infective and non-infective disease states [1]. Most commonly, purpura fulminans affects pediatric patients, and when associated with severe sepsis, purpura fulminans has an in-hospital mortality rate of 42% (or higher) [2]. Typically, the most frequently associated bacterial species found in patient blood cultures with purpura fulminans are group-A Streptococcus (most common in adults), methicillin-susceptible Staphylococcus aureus (MSSA), Streptococcus pneumonia, Vibrio vulnificus, and Neisseria meningitidis (most common in pediatrics) [3-4]. Although MSSA has been reported in several previously documented cases, methicillin-resistant Staphylococcus aureus (MRSA) induced purpura fulminans is an exceedingly rare diagnosis with only a handful of documented cases. Generally, in patients with sepsis-induced purpura fulminans, early goal-directed therapy with broad-spectrum antibiotics and fluid resuscitation is the hallmark of treatment with new research pointing towards the use of activated protein C replacement as an effective adjuvant therapy [4]. Additionally, patients may be treated with fresh frozen plasma (FFP), platelets, intravenous immunoglobulin (IVIg), heparin, and tissue plasminogen activator (tPA).

## Case presentation

Case 1

**Figure 1:**
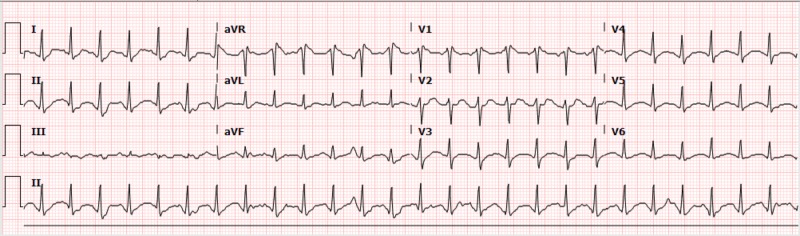
Electrocardiogram on arrival - Case 1

A 54-year-old male was brought in by emergency medical services (EMS), after being found on the floor by his son (unknown down time) with a history significant for frequent falls and alcohol abuse. On EMS arrival to the patient’s home, he was noted to have tachycardia with a rate in the 170s (given multiple rounds of adenosine with no improvement for presumed SVT), fever, and a diffuse erythematous rash (Figure 1). A sepsis alert was called and the patient was transported to the emergency department (ED) for further evaluation and management. On arrival to the ED, the patient was noted to have a temperature of 104.7 (rectally), tachycardia with a rate in the 160s, a blood pressure of 122/85, and a respiratory rate of 22, saturating 99% on 15L via non-rebreather mask. Physical exam revealed a pale, older than stated age male- awake, alert, and oriented to self (answering simple questions appropriately, but unsure of the events leading up to his ED presentation) with diffuse, blanchable, erythematous/purpuric patches throughout his body (primarily throughout the trunk), with non-blanchable patches involved the bilateral lower extremities (no palmar/sole or mucosal involvement). 

Laboratory data, as seen in Table 1, was significant for a platelet count of 13,000/uL, sodium of 125 mmol/L, creatinine of 3.27 mg/dl, total CK of 5,189 units/L, alanine aminotransferase (ALT) of 82 units/L, and aspartate aminotransferase (AST) of 264 units/L (two weeks previously patient was in the ED for an unrelated complaint and had a platelet count of 213,000/uL, sodium of 131 mmol/l, and creatinine of 0.97 mg/dL - liver function studies were not drawn at previous presentation. A working diagnosis of sepsis induced purpura fulminans was established with a consideration for thrombotic thrombocytopenic purpura (TTP) due to patient’s fever, renal failure, altered mental status, and thrombocytopenia. Subsequent laboratory results later confirmed purpura fulminans over TTP with a negative ADAMTS13 testing (A Disintegrin And Metalloproteinase with Thrombospondin type 1 motif, 13), with positive blood cultures that grew MRSA.

**Table 1:** Laboratory data - Case 1 WBC: White blood cells; RBC: Red blood cells; Hgb: Hemoglobin; Hct: Hematocrit; MCV: Mean corpuscular volume; MCH: Mean corpuscular hemoglobin; MCHC: Mean corpuscular hemoglobin concentration; RDW: Red cell distribution width; NRBC: Nucleated red blood cells; PT: Prothrombin time; INR: International normalised ratio; APTT: Activated partial thromboplastin time; AGAP: Anion gap; BUN: Blood urea nitrogen; GFR: Glomerular filtration rate; CKD-EPI: Chronic kidney disease epidemiology collaboration; A/G: Albumin/Globulin; Alk Phos: Alkaline phosphatase; ALT: Alanine aminotransferase; AST: Aspartate aminotransferase; CK: Creatine kinase; ABO, Rh: Blood group A, B, O; Rhesus factor.

Detail	Value w/Units	Normal Range
WBC	6.80 10*3/uL	4.40-10.50
RBC	5.11 10*6/uL	4.00-5.65
Hgb	16.1 g/dL	12.6-16.7
Hct	44.20%	36.9-48.5
MCV	86.5 fL	82.4-99.3
MCH	31.5 pg	27.5-34.1
MCHC	36.4 g/dL	31.7-36.1
RDW	13.80%	11.4-14.9
Platelet Count	13 10*3/uL	139-361
Neutrophils	69.00%	50.0-70.0
Bands	12.00%	0-12
Lymphocytes	1.00%	20.5-45.0
Monocytes	13.00%	1-15
Eosinophils	0.00%	0-5
Basophils	0.00%	0-2
Abs Neutrophil Cnt	4.69 10*3/uL	1.50-7.50
Abs Band Cnt	0.82 10*3/uL	0.00-0.70
Abs Lymphocyte Cnt	0.07 10*3/uL	1.00-4.80
Abs Monocyte Cnt	0.88 10*3/uL	0.00-0.80
Abs Eosinophil Cnt	0.00 10*3/uL	0.00-0.50
Abs Basophil Cnt	0.00 10*3/uL	0.00-0.20
NRBC	1.0 /100{WBCs}	
Atyp Lymphs	3.00%	0-3
Metamyelocytes	2.00%	
Poikilocytosis	1+	
Anisocytosis	1+	
Burr Cells	2+ %	
Dacrocytes	1+	
Toxic Granulation	1+	
Dohle Bodies	1+	
Large Plts	1+	
PT	14.6 s	11.5-14.9
INR	1.13	0.8-1.2
APTT	46.4 s	22.0-38.0
Sodium Lvl	125 mmol/L	135-145
Potassium Lvl	3.1 mmol/L	3.5-5.0
Chloride Lvl	89 mmol/L	98-110
CO2 Lvl	15 mmol/L	24-32
AGAP	21 mmol/L	5-15
Glucose Lvl	99 mg/dL	70-100
BUN Lvl	60 mg/dL	5-15
Creatinine Lvl	3.27 mg/dL	0.60-1.20
GFR Non Afr Amer by CKD-EPI	20 mL/min/{1.73_m2}	
Calcium Lvl	7.4 mg/dL	8.5-10.5
Total Protein Lvl	5.8 g/dL	6.5-8.0
Albumin Lvl	2.4 g/dL	3.2-5.5
Globulin Lvl	3.4 g/dL	1.9-3.9
A/G Ratio	0.7	1.1-2.2
Bilirubin Total	1.2 mg/dL	0.1-1.5
Alk Phos	88 units/L	14-127
ALT	82 units/L	4-51
AST	264 units/L	4-46
CK Total	5,189 units/L	24-200
Ethanol Lvl	<10 mg/dL	
ABO, Rh	O POSITIVE	
Antibody Screen	NEGATIVE	

After initial stabilization and treatment in the ED, the patient was admitted to the intensive care unite (ICU) due to his impending clinical course (deterioration was expected due to patient’s comorbid alcohol abuse and underlying liver disorder). The patient was subsequently intubated and started on vasopressors 15 hours and 19 hours after presentation respectively. Despite aggressive management in the ICU, the patient developed worsening skin discoloration, lost pulses in the extremities, and family withdrew care resulting in death approximately 48 hours after presentation.

Case 2

A 20-year-old female presented to the ED with a complaint of shortness of breath, cough, diarrhea, and fever over the last three days. She also relayed cramping sensations to the upper and lower extremities. The patient was a college student who had travelled to the United States from Columbia four months prior. In the ED, her boyfriend relayed that he recently had the same flu-like symptoms and recovered without any medical treatment. The patient denied any past medical or surgical history. 

In the ED, she was found to have initial vital signs of temperature 97.6, heart rate of 118, respiratory rate of 28, and 88% oxygen saturation on 4L nasal cannula. Physical exam showed an ill, pale appearing young female. Her skin was cool and a mottled rash was clearly visible, mainly in her hands and lower extremities. She had tachypnea, but her lungs were clear to auscultation with symmetrical expansion. She had tachycardia with severely delayed capillary refill. She had a soft abdomen with normal bowel sounds and no tenderness or rebound. Neurologically she was alert and oriented with no motor or speech deficit. 

A full sepsis evaluation was initiated. This included complete blood count (CBC), complete metabolic panel (CMP), prothrombin time (PT), partial thromboplastin time (PTT), international normalized ratio (INR), troponin along with blood cultures, and urinalysis. Arterial blood gas (ABG), creatinine kinase (CK) total, and fibrinogen were also ordered. Computed tomography (CT) imaging of the abdomen and pelvis was ordered to evaluate for pathology causing nausea and diarrhea she had been experiencing. 

Her blood work was significant for bandemia (33%), platelet count of 23,000/uL, an INR of 2.72, creatinine of 2.44 mg/dL, AST 72 units/L, CK total of 892 units/L and a fibrinogen of 66 mg/dL (Table 2). Arterial blood gas showed a pH of 7.264. Lactic acid of 9.7 mmol/L. Her white blood count (WBC) was 5,160/uL, hemoglobin 14.2 g/dL, hematocrit 46.3%, blood urea nitrogen of 25mg/dL. The patients abdominal CT showed nonspecific periportal and pericholecystic edema. Free fluid was noted in the pelvis. The study was technically difficult due to patients thin body habitus and lack of body fat.

**Table 2:** Laboratory data - Case 2 WBC: White blood cells; RBC: Red blood cells; Hgb: Hemoglobin; Hct: Hematocrit; MCV: Mean corpuscular volume; MCH: Mean corpuscular hemoglobin; MCHC: Mean corpuscular hemoglobin concentration; RDW: Red cell distribution width; NRBC: Nucleated red blood cells; PT: Prothrombin time; INR: International normalised ratio; APTT: Activated partial thromboplastin time; AGAP: Anion gap; BUN: Blood urea nitrogen; GFR: Glomerular filtration rate; CKD-EPI: Chronic kidney disease epidemiology collaboration; A/G: Albumin/Globulin; Alk Phos: Alkaline phosphatase; ALT: Alanine aminotransferase; AST: Aspartate aminotransferase; CK: Creatine kinase; ABO, Rh: Blood group A, B, O; Rhesus factor.

Detail	Value w/Units	Normal Range
WBC	5.16 10*3/uL	4.40-10.50
RBC	5.47 10*6/uL	3.75-5.00
Hgb	14.2 g/dL	11.4-14.7
Hct	0.463	34.3-45.5
MCV	84.6 fL	80.5-99.8
MCH	26.0 pg	26.8-33.0
MCHC	30.7 g/dL	31.0-35.4
RDW	0.167	11.7-14.7
Platelet Count	23 10*3/uL	139-361
MPV	10.9 fL	9.7-12.5
Neutrophils	0.39	50.0-70.0
Bands	0.33	0-12
Lymphocytes	0.21	20.5-45.0
Monocytes	0	1-15
Eosinophils	0.01	0-5
Basophils	0.01	0-2
Abs Neutrophil Cnt	2.01 10*3/uL	1.50-7.50
Abs Band Cnt	1.70 10*3/uL	0.00-0.70
Abs Lymphocyte Cnt	1.08 10*3/uL	1.00-4.80
Abs Monocyte Cnt	0.00 10*3/uL	0.00-0.80
Abs Eosinophil Cnt	0.05 10*3/uL	0.00-0.50
Abs Basophil Cnt	0.05 10*3/uL	0.00-0.20
Atyp Lymphs	0.01	0-3
Metamyelocytes	0.03	
Myelocytes	0.01	
Platelet Morphology	NORMAL	
Poikilocytosis	1+	NEGATIVE
Anisocytosis	1+	NEGATIVE
Microcytes	1+	NEGATIVE
PT	29.1 s	11.5-14.9
INR	2.72	0.8-1.2
APTT	102.3 s	22.0-38.0
Fibrinogen Lvl	66 mg/dL	188-468
Sodium Lvl	134 mmol/L	135-145
Potassium Lvl	4.1 mmol/L	3.5-5.0
Chloride Lvl	91 mmol/L	98-110
CO2 Lvl	15 mmol/L	24-32
AGAP	28 mmol/L	5-15
Glucose Lvl	86 mg/dL	70-100
BUN Lvl	25 mg/dL	5-25
Creatinine Lvl	2.44 mg/dL	0.60-1.20
GFR Non Afr Amer by CKD-EPI	28 mL/min/{1.73_m2}	
GFR African Amer by CKD-EPI	32 mL/min/{1.73_m2}	
Calcium Lvl	8.7 mg/dL	8.5-10.5
Magnesium Lvl	1.3 mg/dL	1.5-2.5
Total Protein Lvl	5.9 g/dL	6.5-8.0
Albumin Lvl	3.7 g/dL	3.2-5.5
Globulin Lvl	2.2 g/dL	1.9-3.9
A/G Ratio	1.7	1.1-2.2
Bilirubin Total	1.6 mg/dL	0.1-1.5
Alk Phos	90 units/L	35-104
ALT	29 units/L	5-25
AST	72 units/L	16923
LD	416 units/L	60-200
CK Total	892 units/L	24-200
CK MB	7.1 ng/mL	1.0-6.7
Troponin-T	<0.01 ng/mL	
Troponin-T Interp	NEGATIVE	NEGATIVE
Lipase Lvl	28 units/L	10-60
Beta hCG Qual	NEGATIVE	NEGATIVE

Broad-spectrum antibiotic coverage with vancomycin and pipercillin/tazobactam were given with a 30 cc/kg bolus of normal saline as per sepsis protocol. Lumbar puncture was not performed secondary to low platelets. Due to the patients systemic inflammatory response, bandemia, and abnormal laboratory values, she was admitted to the ICU. Working diagnosis due to the fibrinogen and platelet abnormalities included TTP, hemolytic uremic syndrome, sepsis (possibly from a gastrointestinal source). The ICU team planned to consult hematology and order additional labs.

Once admitted to the ICU, the patient was given 4 units of FFP at the recommendation of hematology and a peripheral blood smear was performed. No schistocytes were noted. Disseminated intravascular coagulation secondary to sepsis was the suspected source of her coagulopathy and infectious disease was consulted.

The infectious disease physician consulted suspected the patient to have purpura fulminans with possible Meningococcemia, Pneumococcemia, Staphylococcemia, Capnocytophaga, or Rickettsial disease. In the ICU, the patient had continued to exhibit signs of septic shock requiring Norepinephrine(Levophed®) to correct hypotension. Antibiotics were switched to Meropenem at that time. Blood cultures returned showing gram-negative diplococci and the diagnosis of meningococcemia was confirmed. At this time the patient’s antibiotics were switched to ceftriaxone 2 gm every 12 hours. Hematology began giving protein C concentrate at this time. The patient continued treatment with ceftriaxone while in the hospital for 10 days before being discharged home.

## Discussion

Purpura fulminans is an external demonstration of serious underlying internal systemic infection. Disseminated intravascular coagulation is the paradoxical phenomenon of clot formation within blood vessels consuming coagulation factors that consequently leads to widespread bleeding. This deregulation within hemostasis is the mechanism behind the dermatologic manifestation of purpura fulminans. 

Protein C is an anticoagulation factor produced in the liver. It is one of several Vitamin K dependent coagulation factors including Factors II, VII, IX, X, protein S, and protein Z. Protein C acts as a natural anticoagulant, disabling factors Va and VIIIa when functioning in its activated form. Patients can have both an inherited protein C deficiency, or an acquired deficiency as described in the preceding cases.

Meningococcemia and infections secondary to Staphylococcus aureus lead to remarkably reduced levels of activated protein C due to dysfunction of the endothelial protein C activation pathway. In addition to activated protein C’s anticoagulation profile, it also has a role in inflammatory response modulation. Administration of protein C concentrate reduces skin tissue damage, preventing irreparable skin damage, and decreases the inflammatory cascade [5].

Treatment

In patients with sepsis induced purpura fulminans, early goal directed therapy with broad spectrum antibiotics and fluid resuscitation is a hallmark of treatment with new research pointing towards the use of activated protein C replacement (via protein C concentrate) as an effective adjuvant therapy [5]. When given early in the disease course, protein C concentrate may in fact reduce both morbidity and mortality [3]. Dosing of protein C concentrate is 100-120 IU/kg, with 3 subsequent doses of 60-80 IU/kg q6hrs, as well as a maintenance dose of 45-60 IU/kg q6-12hrs [6]. Additionally, patients may be treated with FFP (in the setting of purpura fulminans secondary to protein c defiency), platelets, IVIG, heparin, and tPA with varying degrees of improvement/success dependent upon the clinical presentation of purpura fulminans (anticoagulation is not currently recommended for treatment of purpura fulminans when caused by, or in the presence of sepsis).

Regarding platelets, traditional treatment guidelines of platelet replacement is indicated for a platelet count <50,000 in the setting of active bleeding and <10,000 without active bleeding but in purpura fulminans this may not be effective. Alternatively, replacing platelets for any level <20,000 without active bleeding has been recommended (treatment in the setting of active bleeding is unchanged). If there is concern for TTP, platelets are classically withheld, as there have been reported cases of myocardial infarctions and stroke following administration of platelets. No formal studies have shown statistically significant negative effects of platelet transfusion in this patient population but due to these previous reports FFP is administered in place of platelets [7].

## Conclusions

Purpura fulminans is most commonly caused by meningococcemia in pediatrics and Group-A Streptococcus in adults but can also be caused by staphylococcus (typically MSSA), Vibrio vulnificus, pneumococcus, and non-infectious states including protein C and S deficiencies. MRSA induced purpura fulminans is an exceedingly rare diagnosis.

Protein C concentrate can, and should be, considered in patients with suspected purpura fulminans in the setting of severe sepsis in consultation with hematology as it has shown improvement in morbidity and mortality. Platelets should be transfused for platelet counts <20,000 in the setting severe sepsis induced purpura fulminans without active bleeding. Treatment for purpura fulminans should always initially be directed towards the underlying cause of purpura fulminans with broad-spectrum antibiotics and fluid resuscitation.
